# Myricanol 5-fluorobenzyloxy ether regulation of survivin pathway inhibits human lung adenocarcinoma A549 cells growth in vitro

**DOI:** 10.1186/s12906-020-03062-8

**Published:** 2020-09-03

**Authors:** Guan-hai Dai, Xuan Chen, Ze-ming Ren, Chen-jie Dai, Ye-ling Tong, Ke-qun Chai

**Affiliations:** 1 Zhejiang Academy of Traditional Chinese Medicine, Institute of Basic Medicine, Hangzhou, 310007 China; 2 Zhejiang University-University of Edinburgh Institute, Zhejiang University, Haining, 314400 China; 3 Oncology Department, Tongde Hospital of Zhejiang Province, Hangzhou, 310012 China

**Keywords:** Myricanol 5-fluorobenzyloxy ether, Antitumor, Human lung adenocarcinoma A549, Survivin pathway, Cell growth inhibition, Mitochondrial membrane potential

## Abstract

**Background:**

This study aimed to explore the growth inhibitory effect of myricanol 5-fluorobenzyloxy ether (5FEM) and its underlying mechanisms in human lung adenocarcinoma A549 cells in vitro.

**Methods:**

5FEM was obtained by the chemical modification of myricanol with fluorobenzyloxy ether at the OH(5) position. The cytotoxicity, cell apoptosis, cell cycle, mitochondrial membrane potential (ΔΨm), scratch test, colony formation, and the expression levels of the key survivin pathway-related genes in A549 were evaluated.

**Results:**

5FEM could significantly inhibit A549 cell growth; induce cell apoptosis; increase G0/G1 population; reduce ΔΨm; inhibit cell migration and colony formation; upregulate caspase-9, P21, and Bax expression levels; and downregulate PARP, survivin, and Bcl-2 expression level.

**Conclusion:**

These results enhanced our understanding of 5FEM and aid the discovery of novel myricanol derivatives as potential antitumor agents.

## Background

Lung cancer is one of the most common malignant tumors and has the highest incidence and mortality. Its 5-year relative survival rate is generally < 15% [1, 2]. Lung cancer is divided into non-small cell lung cancer (NSCLC) and small cell lung cancer (SCLC), with the former accounting for 85% of all patients. The occurrence and development of NSCLC are closely related to external factors, such as smoking, air pollution, and genetic factors. Despite the availability of chemotherapy regimens, the mortality rate of NSCLC has not decreased [3]. Thus, the invasion and metastatic mechanisms in NSCLC should be understood to improve treatment individualization.

Inhibitor of apoptosis protein (IAP) family regulates apoptosis. As a new member of the recently discovered IAP family, survivin plays an important role in cell apoptosis and cell cycle regulation [4]. In 1997, Ambrosini et al. [5] firstly isolated survivin from the human genome library using effect cell protease receptor 1 cDNA and found that it is still the strongest apoptotic inhibitor protein. The gene is located on human chromosome 17q25, has a full length of ~ 14.5 kb, contains four exons and three introns, and encodes a survivin protein with a molecular weight of 16.3 kD and contains 142 amino acids [6]. Survivin inhibits cell apoptosis, participates in mitosis and cytokinesis regulation, and has a strict cell cycle-dependent expression [7]. Survivin inhibits apoptosis by specifically binding to caspase-3 and -7, which are the downstream terminal effectors of apoptotic pathway, and subsequently inactivating them [8]. Survivin has been recognized as a tumor gene because it is highly expressed in cancer tissues, while rarely expressed in normal tissues [9]. Several tumors are resistant to drugs, and an important factor in this resistance is the high survivin expression level [10]. Therefore, reducing the expression of survivin provides a starting point in weakening the drug resistance of cancer cells. Chen et al. reported that downregulation of survivin expression by RNA interference can inhibit cell proliferation, significantly reduce cell invasion and metastasis, and effectively improve the sensitivity of radiotherapy and chemotherapy to cancer cell apoptosis [11].

Since survivin is highly expressed in cancer and low in normal tissues, targeted survivin therapy to treat cancer may have less damage to normal tissues. This protein also has a strong ability to inhibit apoptosis and thus has been regarded as a new important target of cancer treatment with good application prospect.

*Myrica rubra* (Lour.) Sieb. Et Zucc. is a myricaceae Myrica plant and a subtropical fruit tree found in China and other Asian countries [12, 13]. Myricanol was extracted from *M. rubra* bark through system solvent extraction [14]. Considering its unique chemical structure, myricanol has attracted widespread attention of phytochemistry and pharmacy researchers worldwide. At present, the reports on the pharmacological activities of myricanol mainly involve anti-inflammatory, antiviral, antioxidant, and scavenging activities [15–17], but its huge antitumor potential has not yet been studied.

Myricanol remarkably inhibits the proliferation of human lung adenocarcinoma (A549), hepatoma (HepG2), and human promyelocytic leukemia (HL-60) cells [18, 19]. Through a series of experiments, we observed that the novel myricanol derivatives with stronger antitumor activity and low toxicity to normal cells. As a result, 5-Fluorobenzyloxy ether (5FEM) is more potent than myricanol and other derivatives [20].

In this study, the 5FEM regulation of the survivin pathway inhibiting human lung adenocarcinoma A549 cell growth in vitro was investigated.

## Methods

### Chemicals and reagents

5-FEM (98.2% purity, batch number: 20170628) was prepared as previously described [20], Myricanol was added to acetone in a round-bottom flask, and stirred until dissolved. K_2_CO_3_ was added, and then 4-fluorine benzyl bromide was added in the absence of light at room temperature. The solvent was evaporated using a rotary evaporator. The chromatography products were collected, combined, and spin dried. 5FEM was obtained as a yellow solid. 5FEM at 200 mM was dissolved completely in dimethyl sulfoxide (DMSO) to obtain a stock solution, which was stored at − 20 °C and diluted with a medium before use. Tritiated thymidine (^3^H-TdR, 1 mci/ml, 37 MBq) was purchased from China Isotope & Radiation Corporation (Beijing, China, product code: NET027L001MC, Lot: 201807). YM155 (C_20_H_19_BrN_4_O_3_) was purchased from MedChemExpress (Cat. No.: HY-10194/CS-0336, Lot: 24444). Puromycin dihydrochloriede (> 99% purity) was purchased from MDBio Inc. (CAS: 58–58-2, Lot: H5160501). Annexin V PE/7-Amino-Actinomycin D (7AAD) kit was purchased from Life Science Reagent Biotechnology (batch number: 73241151). Mitochondrial Membrane Potential Assay Kit (with JC-1, C_20_H_19_BrN_4_O_3_) was purchased from Beyotime Biotechnology (Shanghai, China, product code: C2006, Lot: 022618180502). Fetal bovine serum (FBS) was purchased from ZheJiang Tianhang Biological Co., Ltd. (Hangzhou, Zhejiang, China, product code: 11011–8611, Lot: 20171011). Culture reagents, including phosphate-buffered saline (PBS, product code: GNM20012, Lot: 1902270107), RPMI-1640 medium (product code: GNM31800-S, Lot: 190506030), and 0.25% trypsin (product code: GNM25200, Lot: 1810190406), were purchased from Gino Biological Pharmaceutical Technology Co., Ltd. (Hangzhou, Zhejiang, China). PrimeScript™ RT Reagent Kit were purchased from TaKaRa Bio Inc. (Japan, Cat: RR037A, Lot: AK5601). SYBR Premix EX Taq™IIwere purchased from TaKaRa Bio Inc. (Japan, Cat: RR820A, Lot: AJ61452A) and primary rabbit monoclonal antibodies against caspase-9, Bax, Bcl-2, Cyt C, PARP, survivin, p21, and actin were purchased from Proteintech Group, Inc. Rosement (IL 60018, USA). All other chemicals used were of analytical grade and purchased from Sigma-Aldrich (Shanghai, China).

### Construction of survivin-overexpressing A549-homo BIRC5 cell line

The human lung carcinoma A549 cell line was obtained from the cell bank of Chinese Academy of Sciences (Shanghai, China). The target gene was obtained by whole gene synthesis. The target vector was digested by endonuclease. The purified polymerase chain reaction (PCR) product was linked to the linearized vector and transformed into bacterial competent cells. The clones were identified by restriction enzyme digestion, which proved that the target gene had been directionally linked to the target vector. Then, the positive clones were sequenced and analyzed, and the correct comparison was to construct a successful target gene expression plasmid vector. The constructed lentivirus overexpression plasmid vector (LV5-Homo BIRC5) was extracted by ultrapure endotoxin removal.

Transfection experiment: Human lung adenocarcinoma A549 cell line at logarithmic growth stage was digested with 0.1% trypsin and made into 4 × 10^4^/ml single cell suspension. A total of 100 μl of suspension were added to 96-well flat-bottomed culture plate and cultured at 37 °C in a humidified atmosphere of 5% CO_2_. Cell supernatant was removed the next day, and 90 μl of medium containing 5 μg/ml of polybrene was added. Then, LV5-Homo BIRC5 with 10× dilution was added. LV5NC control and culture medium blank control were made at the same time. The virus solution was removed after 24 h incubation and replaced with fresh complete culture medium. The fluorescence expression was observed after 72 h of infection. At the same time, puromycin medium containing 0.6 μg/ml was used in screening culture. After extended culture, survivin expression was detected by PCR.

### Cell culture

A549 and A549-Homo BIRC5 cells was cultured in RPMI-1640 medium supplemented with 10% fetal bovine serum (FBS) and 1% penicillin and streptomycin solution (100 U/mL penicillin and 100 μg/mL streptomycin). Cells were cultured at 37 °C in a 5% CO_2_ humidified incubator and were split regularly before they attained approximately 80% confluence.

### Cell cytotoxicity assay

Cell cytotoxic was measured using the ^3^H-TdR assay. Briefly, the A549-Homo BIRC5 cell (5 × 10^3^/well) at logarithmic growth stage was seeded in 96-well flat-bottomed culture plate (Cornin Life Sciences, Corning, NY, USA) and incubated at 37 °C with 5% CO_2_ for 24 h before treated. A549-Homo BIRC5 cells were divided into 3 groups: 5FEM group (addition of 5FEM, 3.12–100 μM), negative control group (addition of DMSO, 0.1%), and positive control group (addition of YM155, 10 nM). After respectively treatment for 48 h, ^3^H-TdR (0.5 μci/50 μL in medium) was added to each well and incubated for 16 h. The cells were collected (D961962 Cell Collector, Perkin Elmer, USA), and the counts per min for each sample were determined by liquid scintillation counting (Micro Beta 2450, Perkin Elmer, USA). Cell growth inhibitory effect of 5FEM was assessed. The experiments were performed independently in triplicate.

### Cell apoptosis analysis

A549-Homo BIRC5 cell (5 × 10^3^/well) at the logarithmic growth stage was seeded in six-well plates for 24 h. The cells were treated with 5FEM of different concentrations (2.5, 5.0, and 10.0 μM), YM155 (10 nM), or vehicles (0.1% DMSO) for 48 h. The rate of apoptosis was determined using Annexin V-PE / 7AAD staining by FCM (Beckman Coulter, Model EPICS XL-4). The experiments were performed independently in triplicate.

### Cell cycle analysis by FCM

A549-Homo BIRC5 cell (5 × 10^3^/well) was treated the same as apoptosis analysis. The cells (1 × 10^6^) cells were washed with ice-cold PBS, fixed with 75% (v/v) ethanol for 4 h at 4 °C. Then cells were stained with PI/RNase. Cell cycle analysis was performed using a FCM (Beckman Coulter, Model EPICS XL-4). The experiments were performed independently in triplicate.

### ΔΨm determination

According to the manufacturer’s protocol, the mitochondrial membrane potential (ΔΨm) assay kit (KeyGEN Biotech, Nanjing, China) with JC-1 was used to detect the ΔΨm. A549 cell was treated the same as apoptosis analysis. The cells grown on glass coverslips were cultured with 5 μg/mL JC-1 at 37 °C for 20 min. After staining, the cells were rinsed with JC-1 staining buffer three times. Next, flow cytometry (Beckman Coulter, Model EPICS XL-4) was used to determine the fluorescence intensity. Afterward, the results of all analyses were carried out using FlowJo v7.6 software (Tree Star Inc., Ashland, OR). The experiments were performed independently in triplicate.

### Scratch test for cell migration

A horizontal line with marker pen was drawn approximately every 0.5–1.0 cm across the well on the back of the six-well plates. Each well passed through at least five lines that were uniform and parallel. A549-Homo BIRC5 cell (3 × 10^5^/well) at the logarithmic growth stage was seeded in six-well plates for 24 h. Scratch along the ruler with the pipette tips perpendicular to the back of the horizontal line. The cells were washed three times with PBS, and the underlying cells were removed. The cells were treated with different concentrations of 5FEM (2.5, 5.0, and 10.0 μM), YM155 (10 nM) or vehicle (0.1% DMSO). The FBS concentration in the experimental medium was 2%. The width of the scratches was observed cultured at 37 °C in a humidified atmosphere of 5% CO_2_ at 0, 24, and 48 h.

Each scratch was measured by five random fields. Relative cell mobility was calculated in each drug group compared with the control. The experiments were performed independently in triplicate.

### Colony formation

A549-Homo BIRC5 cell (200 cells/well) at the logarithmic growth stage was seeded in six-well plates for 24 h. The cells were treated with different concentrations of 5FEM (2.5, 5.0, and 10.0 μM), YM155 (10 nM), or vehicle (0.1% DMSO) cultured at 37 °C in a humidified atmosphere of 5% CO_2_. The culture medium was replaced every 3 to 4 days. After 10 days, the culture medium was discarded, washed with PBS, and stained with crystal violet for 5 min. The number of clones of > 50 cells was counted, and the colony formation rate was calculated.

### Total RNA isolation and quantitative real-time PCR (qPCR) analysis

A549-Homo BIRC5 cells were treated with different concentrations of 5FEM (2.5, 5.0, and 10.0 μM), YM155 (10 nM), or vehicle (0.1% DMSO) for 48 h. The total RNA of each sample was isolated using an EZNA Total RNA Kit (Omega Bio-tech Inc., USA) according to the manufacturer’s protocol. Reverse transcription was performed using a PrimeScript™ RT reagent Kit (TaKaRa, Japan), and 1 μg RNA was reverse transcribed into cDNA in a 25 μL reaction system and stored at − 80 °C until use. All oligonucleotide primers were designed using the Perlprimer software and synthesized commercially (Sangon Biotechnology, Shanghai, China). The sequence of the primers is as follow: 5′-GGTCTCCTCTGACTTCAACA-3′ (forward) and 5′-AGCCAAATTCGTTGTCATAC-3′ (reverse) for GAPDH, 5′-TCTGGAGGATTTGGTGATGTC-3′ (forward) and 5′-CATTTTCTTGGCATCAGGTC-3′ (reverse) for caspase-9,5′-TTAGCAGCGGAACAAGGAGT-3′ (forward) and 5′-AGAAACGGGAACCAGGACAC-3′ (reverse) for p21, 5′-AAGCTGAGCGAGTGTCTCAAG-3′ (forward) and 5′-CAAAGTAGAAAAGGGCGACAAC-3′ (reverse) for Bax, 5′-ATGAAGTGAAGGCCATGATTG-3′ (forward) and 5′-TCCTTTAACGATGTCCACCAG-3′ (reverse) for PARP, 5′-CCCACTGAGAACGAGCCAGA-3′ (forward) and 5′-AAAGGAAAGCGCAACCGGAC-3′ (reverse) for survivin, 5′-ATGTGTGTGGAGAGCGTCAAC-3′ (forward) and 5′-AGAGACAGCCAGGAGAAATCAAAC-3′ (reverse) for Bcl-2. qPCR was performed on the StepOnePlus (Applied Biosystems, USA) using a SYBR Premix EX Taq™ II (TaKaRa, Japan) following the manufacturer’s protocol. The two-step PCR reaction conditions are as follow: initial denaturation at 95 °C for 30 s, followed by 40 cycles of denaturation at 95 °C for 5 s; annealing and extension at 60 °C for 30 s. Each sample was tested in triplicate. The relative fold change in gene expression was calculated as 2^−ΔΔCt^ method [21].

### Western blot analysis

A549-Homo BIRC5 cells were pretreated with different 5FEM (2.5, 5.0, and 10.0 μM), YM155 (10 nM), or vehicle concentrations (0.1% DMSO) for 48 h. The total protein was extracted as previously described,[20] and equal amounts of cellular protein (30 μg) were separated through electrophoresis and then transferred to a nitrocellulose membrane by using an electrotransfer method. After being blocked with nonfat milk, the membranes were incubated with proper primary antibodies against Caspase-9, Bax, Bcl-2, Cyt C, PARP, survivin and p21 (1:800) or Actin (1:2000) at 4 °C overnight. The blots were washed with TBST and incubated with secondary antibody (1:10000) for 1 h at room temperature. The bands were visualized by the ECL method. The Western blot analysis results were evaluated using the Quantity One software.

### Statistical analysis

The results were expressed as means±SE. Statistical comparisons were made with a two-sided *t*-test. *p* < 0.05 was considered significant.

## Results

### Survivin expression level on A549 cells with LV5-homo BIRC5 transfection

**Fig. 1:**
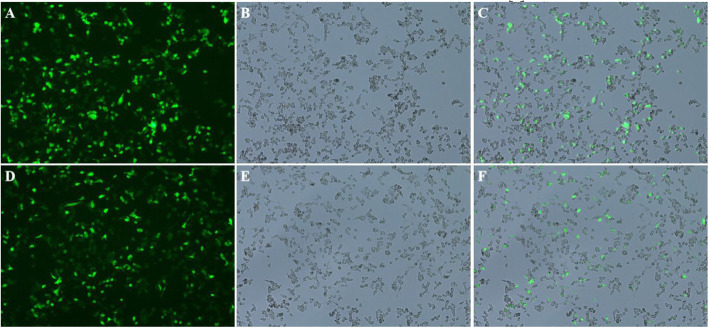
Photographs taken by fluorescence microscopy after transfection. **a**: Photographs of fluorescent labeling after LV5-Homo BIRC5 transfected cells. **b**: Photographs of common light after LV5-Homo BIRC5 transfected cells.. **c**: Photographs of merge after LV5-Homo BIRC5-transfected cells. **d**: Photographs of fluorescent labeling with LV5NC control. **e**: Photographs of common light with LV5NC control. **f**: Photographs of merge with LV5NC control

**Fig. 2:**
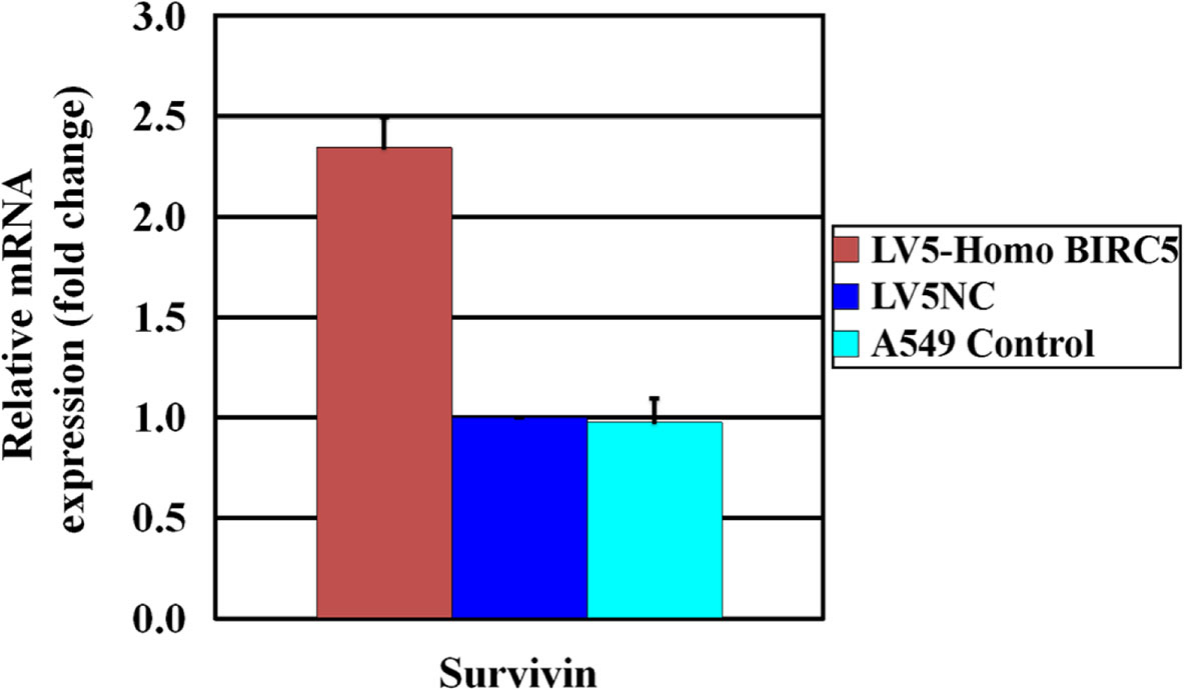
Effects of survivin expression level on A549 cells with LV5-Homo BIRC5 transfection

Human lung adenocarcinoma A549 cell line was transfected with LV5-Homo BIRC5. LV5NC control and culture medium blank control were also setted. At the same time, puromycin medium containing 0.6 μg/ml was used to screen the culture. Photographs were taken by a fluorescence microscope after transfection (Fig. 1). The GFP expression level in A549 cells was observed under fluorescence microscope after transfecting with LV5-Homo BIRC5 and LV5NC. After extended culture, the survivin expression level was detected by qRT-PCR. The results showed that the survivin expression level in A549 cells was increased significantly in LV5-Homo BIRC5 transfection group than control group, but the change between LV5NC-transfected and normal A549 cells was not significant (Fig. 2).

### Cytotoxicity assay

**Fig. 3:**
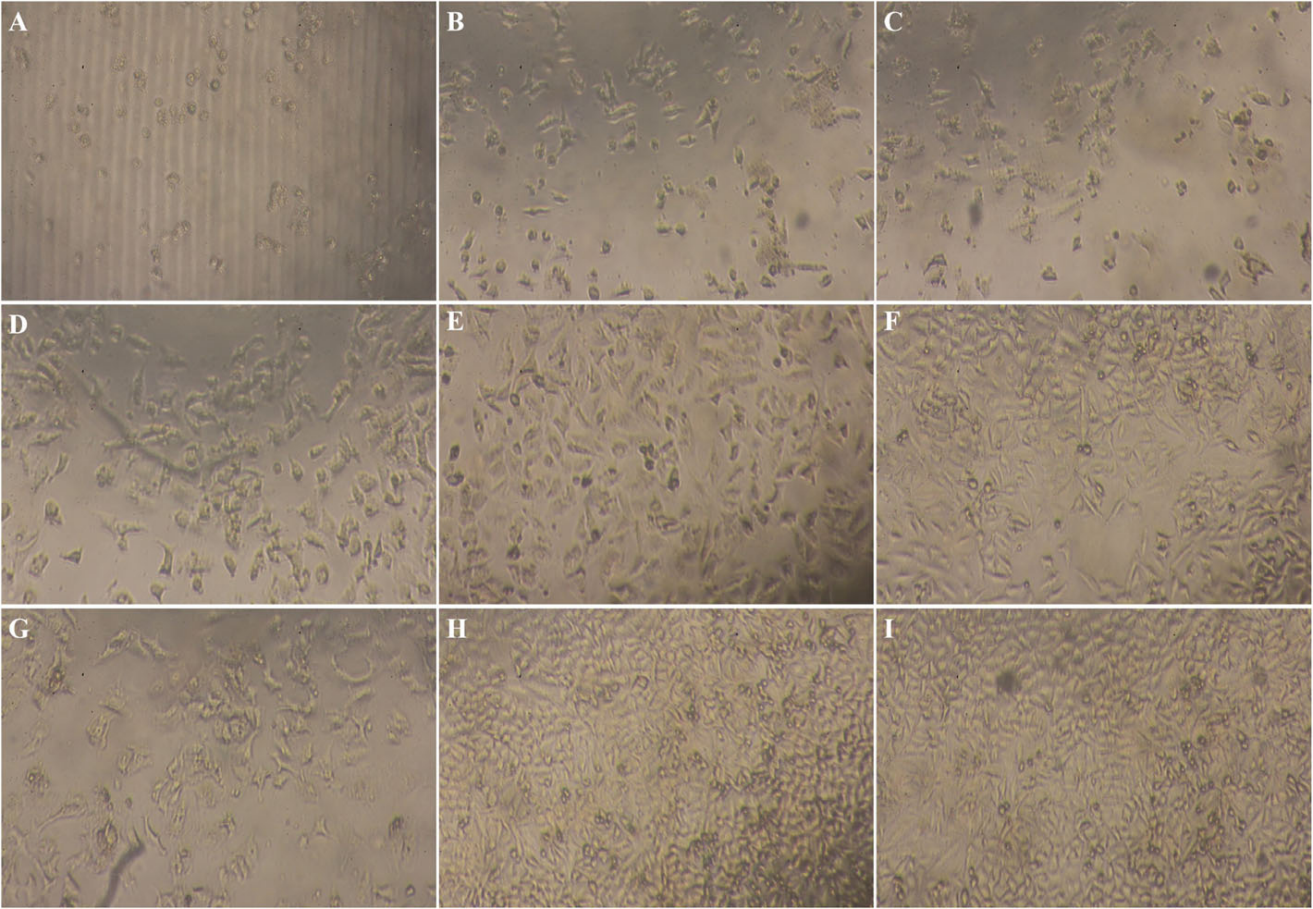
Photographs of growth inhibitory effect of 5FEM on A549-LV5-Homo BIRC5 cells (100×); **a** 100 μM 5FEM, **b** 50 μM 5FEM, **c** 25 μM 5FEM, **d** 12.5 μM 5FEM, **e** 6.25 μM 5FEM, **f** 3.12 μM 5FEM, **g** 10 nM YM155, **h** Vehicle (0.1% DMSO) control, and **i** RPMI 1640 medium control

**Fig. 4:**
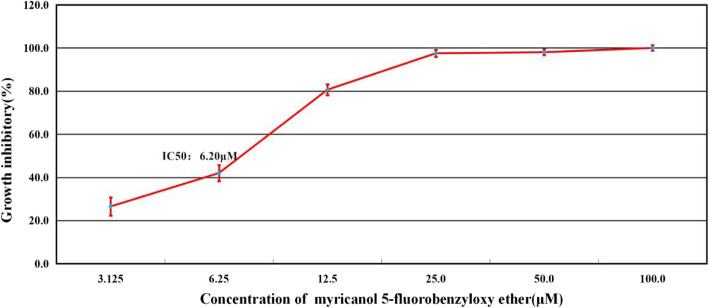
Growth inhibitory effect of 5FEM on A549-LV5-Homo BIRC5 cells after 72 h treatment

The effects of 5FEM on the viability of A549-Homo BIRC5 cells were examined using the ^3^H-TdR assay. After 48 h treatment, 5FEM effectively inhibited the viability of A549-Homo BIRC5 cells (Fig. 3). The number of adherent cells decreased in varying degrees. The cells became round and reduced in size suspended in the culture medium. The granules and vacuoles were present in the cytoplasm, and the number of cells decreased significantly. In the control group, the cells grew adherently, the membrane was intact, the cytoplasm was full, and the adjacent cells grew and fused into pieces. The IC_50_ values of 5FEM on A549-Homo BIRC5 cells were 6.20 ± 0.52 μM (Fig. 4).

### Cell apoptosis assay

**Fig. 5:**
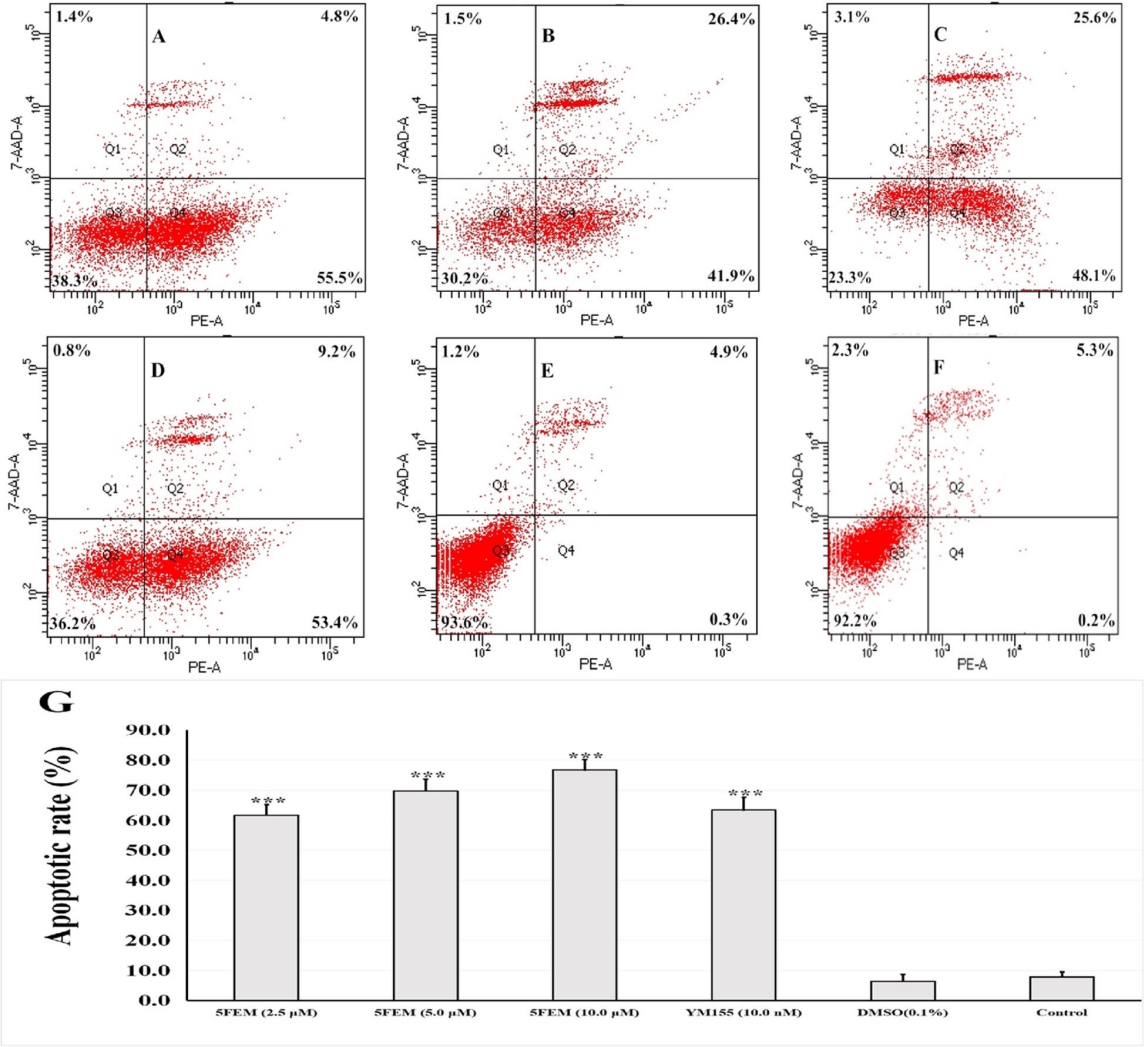
Effects of 5FEM on apoptosis of A549-Homo BIRC5 cells. The cells were treated with different 5FEM concentrations for 48 h and labeled with Annexin V PE and 7AAD; **a** 2.5 μM 5FEM, **b** 5.0 μM 5FEM. **c** 10.0 μM 5FEM. **d** 10.0 nM YM155. **e** Vehicle (0.1% DMSO) control. **f** RPMI 1640 medium control. Annexin−/PI-(LL), viable cells; annexin+/PI-(LR), early apoptosis cells; annexin+/PI+ (UR), late apoptosis cells. LL: lower left; LR: lower right; UR: upper right. **g** Column figure of apoptotic rate in different groups. All data were expressed as mean ± SE of three experiments and each experiment included triplicate repeats. ***p < 0.001 vs. vehicle control

The A549-Homo BIRC5 cells were treated with different 5FEM concentrations (2.5, 5.0, and 10.0 μM) for 48 h. The apoptosis induced by 5FEM was quantified by flow cytometry (FCM) after cell labeling with Annexin V PE and 7AAD (Fig. 5a–f). The apoptotic rate was increased from 15.2% (untreated cells) to 76.7% (drug-treated cells), thereby indicating that 5FEM induced A549-Homo BIRC5 cell apoptosis. The statistics showed that 5FEM can significantly induce apoptosis in A549-Homo BIRC5 cells (Fig. 5g).

### Cell cycle analysis

**Fig. 6:**
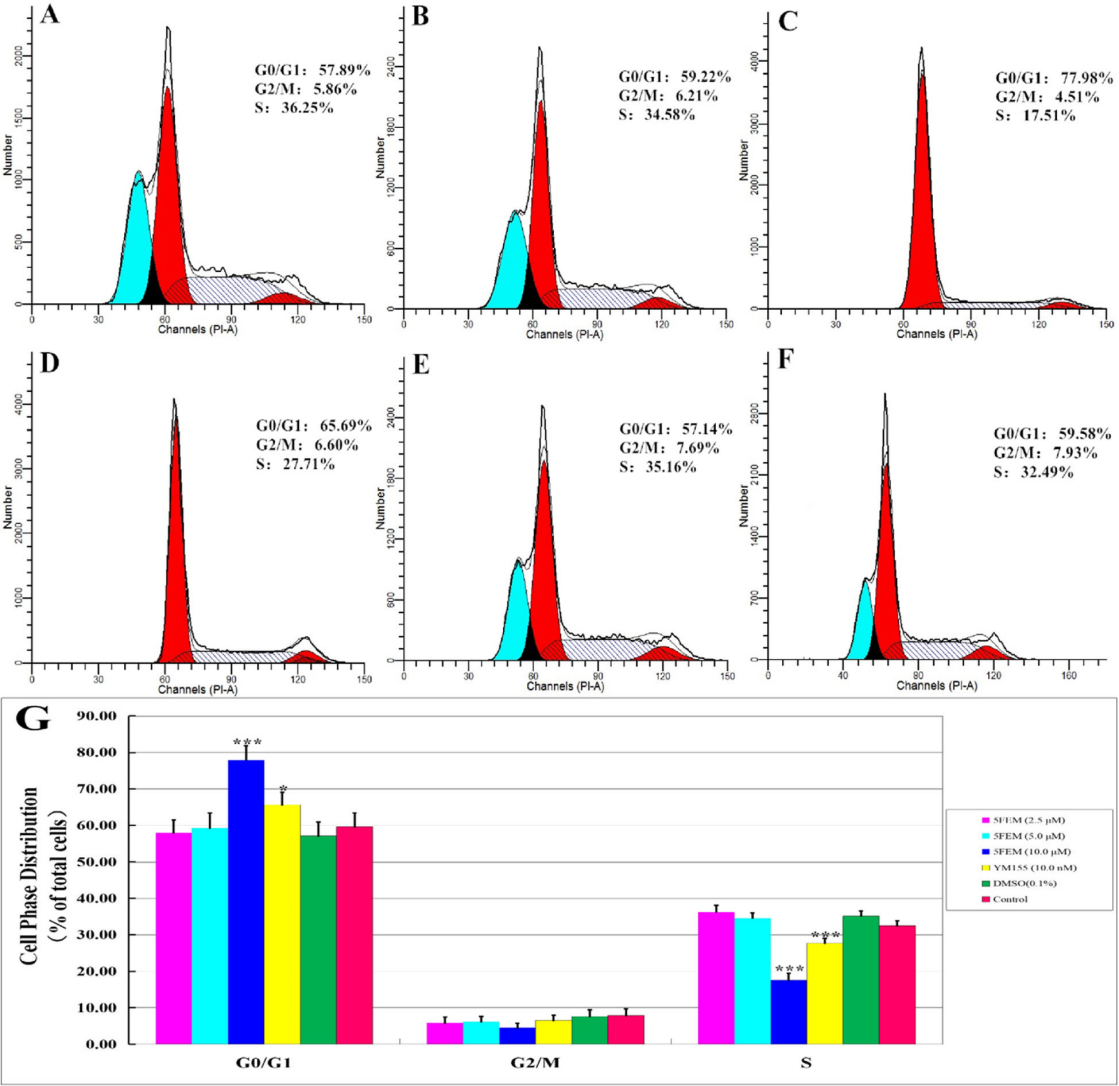
Effects of 5FEM on cell cycle of A549-Homo BIRC5 cells. The cells were treated with different 5FEM concentrations for 48 h and subjected to flow cytometry. The representative DNA fluorescence histogram of PI-stained A549-Homo BIRC5 cells showing the cell cycle distribution; **a** 2.5 μM 5FEM, **b** 5.0 μM 5FEM. **c** 10.0 μM 5FEM. **d** 10.0 nM YM155. **e** Vehicle (0.1% DMSO) control. **f** RPMI 1640 medium control. **g** Column figure of cell cycle rate in different groups. All data were expressed as mean ± SE of three experiments, and each experiment included triplicate repeats. *p < 0.05, ***p < 0.001 vs. vehicle control

To explain the underlying mechanisms of cell growth inhibition, we used FCM with PI staining to analyze the phases of cell cycle of A549-Homo BIRC5 cells treated with different 5FEM concentrations (2.5, 5.0, and 10.0 μM) for 48 h. As shown in Fig. 6a–f, 5FEM caused a concentration-dependent disturbance of the cell cycle distribution. The most pronounced increase was observed in the G0/G1 population, in which the number of cells in the S phase decreased (Fig. 6g).

### Mitochondrial membrane potential (ΔΨm)

**Fig. 7:**
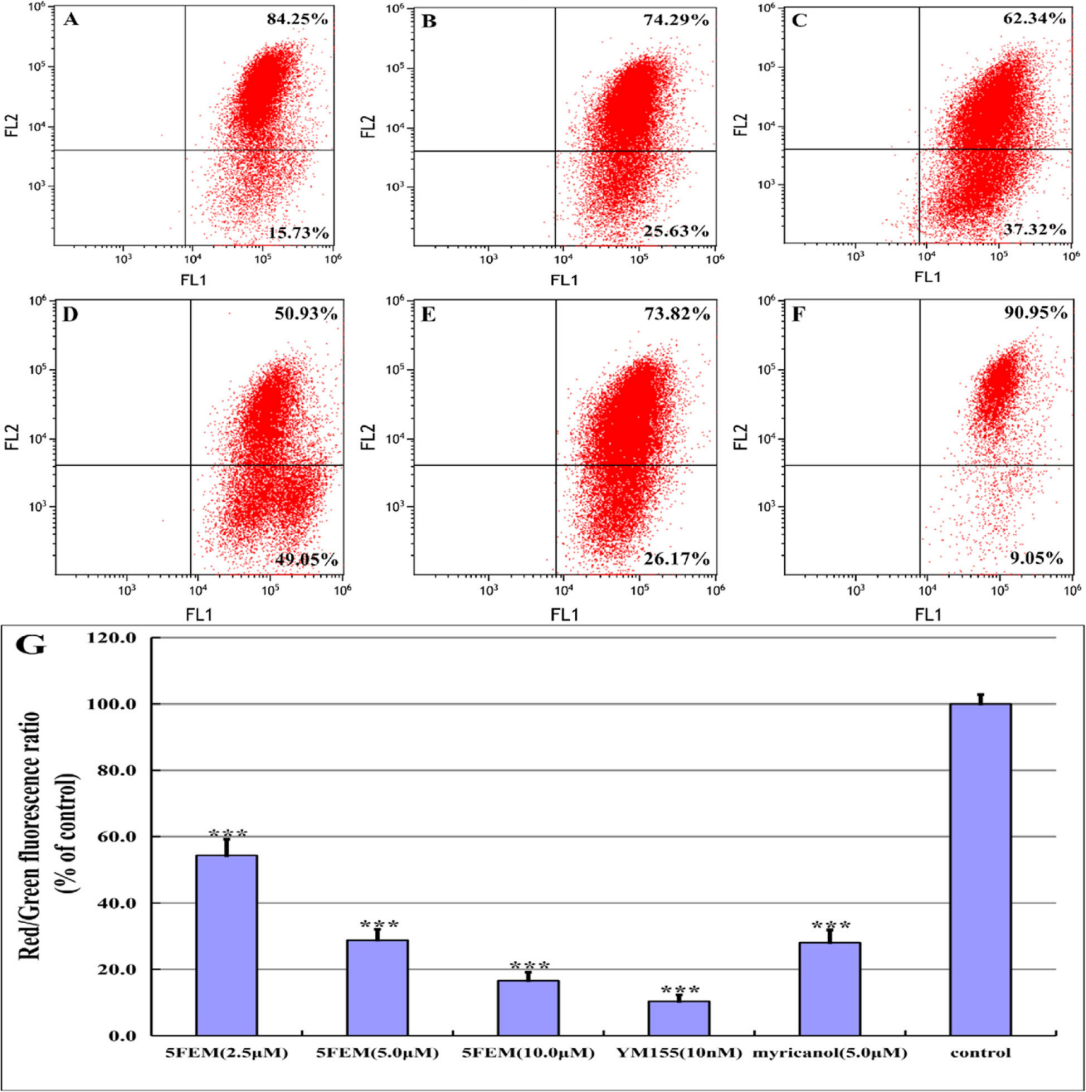
5FEM induces the reduction of mitochondrial membrane potential (ΔΨm) of A549 cells; **a** 2.5 μM 5FEM, **b** 5.0 μM 5FEM, **c** 10.0 μM 5FEM, **d** 10.0 nM YM155, and **e** 5.0 μM myricanol. **f** Vehicle (0.1% DMSO) control. **g** Column figure of ΔΨm in different groups. All data were expressed as mean ± SE of three experiments, and each experiment included triplicate repeats. ***p < 0.001 vs. vehicle control

Considering that mitochondria act as the center of the cell apoptotic progresses, we further evaluated the ΔΨm of 5FEM on A549 cells. As shown in Fig. 7a–g, 5FEM treatment resulted in a significantly high green-to-red fluorescence ratio, thereby indicating that the number of cells with low ΔΨm increased.

### Cell migration

**Fig. 8:**
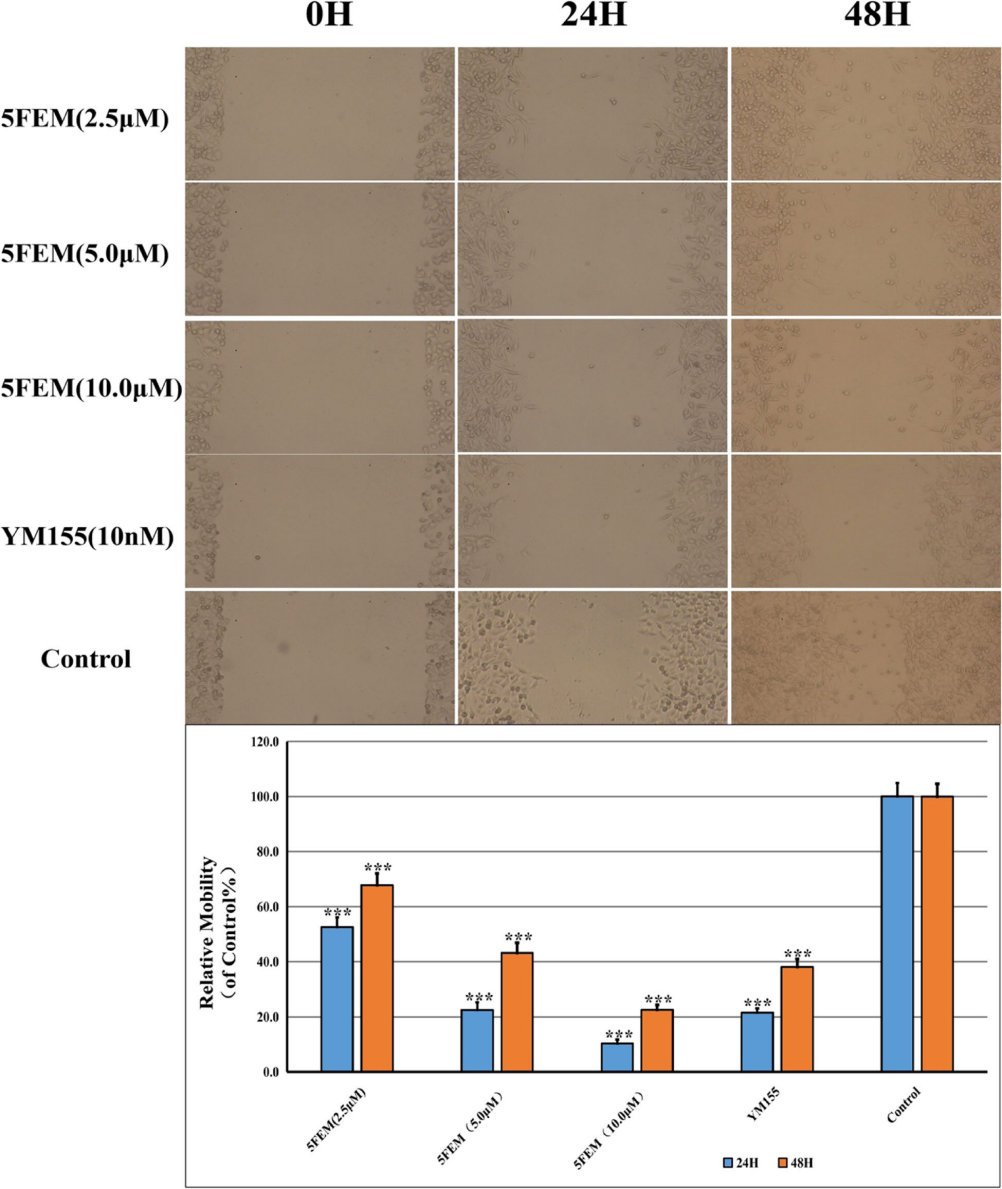
Effects of 5FEM on cell migration of A549-Homo BIRC5 cells. All data were expressed as mean ± SE of three experiments, and each experiment included triplicate repeats. ***p < 0.001 vs. vehicle control

Tumor metastasis and invasion are the most life-threatening aspects of lung cancer. We examined whether 5FEM affects A549-Homo BIRC5 cell migration and invasion abilities. Wound closure area was large after 5FEM administration (2.5, 5.0, and 10.0 μm) by performing wound scratch assay in A549-Homo BIRC5 (Fig. 8). Compared with the control group, the wound width at 24 and 48 h was significantly larger in the treatment group (*p* < 0.001), indicating that 5-fluorobenzyloxymyricetin could significantly inhibit A549-Homo BIRC5 cell migration.

### Cell colony formation

**Fig. 9:**
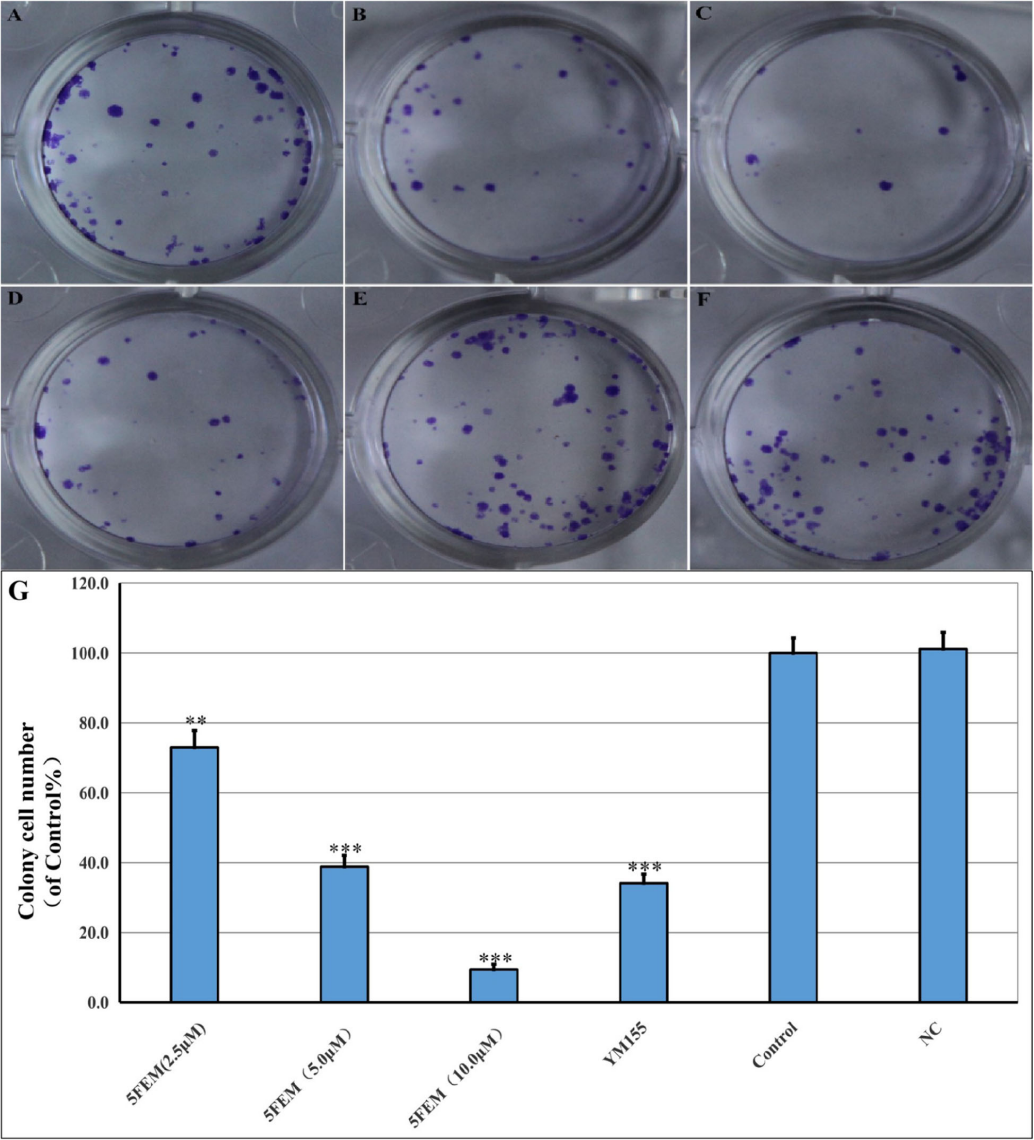
Effects of 5FEM on cell colony formation of A549-Homo BIRC5; **a** 2.5 μM 5FEM, **b** 5.0 μM 5FEM, **c** 10.0 μM 5FEM, and **d** 10.0 nM YM155. **e** Vehicle (0.1% DMSO) control. **f** RPMI 1640 medium control. **g** Column figure of colony formation in different groups. All data were expressed as mean ± SE of three experiments and each experiment included triplicate repeats. **p < 0.01, ***p < 0.001 vs. vehicle control

The effects of 5FEM on A549-Homo BIRC5 cell colony formation was determined by crystal violet staining. As shown in Fig. 9, the size and the number of colonies exhibited a significant decrease after 5FEM administration (2.5, 5.0, and 10.0 μm; Fig. 9). Statistical analysis showed that the number of colonies were considerably fewer than that of the control group (*p* < 0.01 to p < 0.001).

### mRNA expression of survivin-related genes

**Fig. 10:**
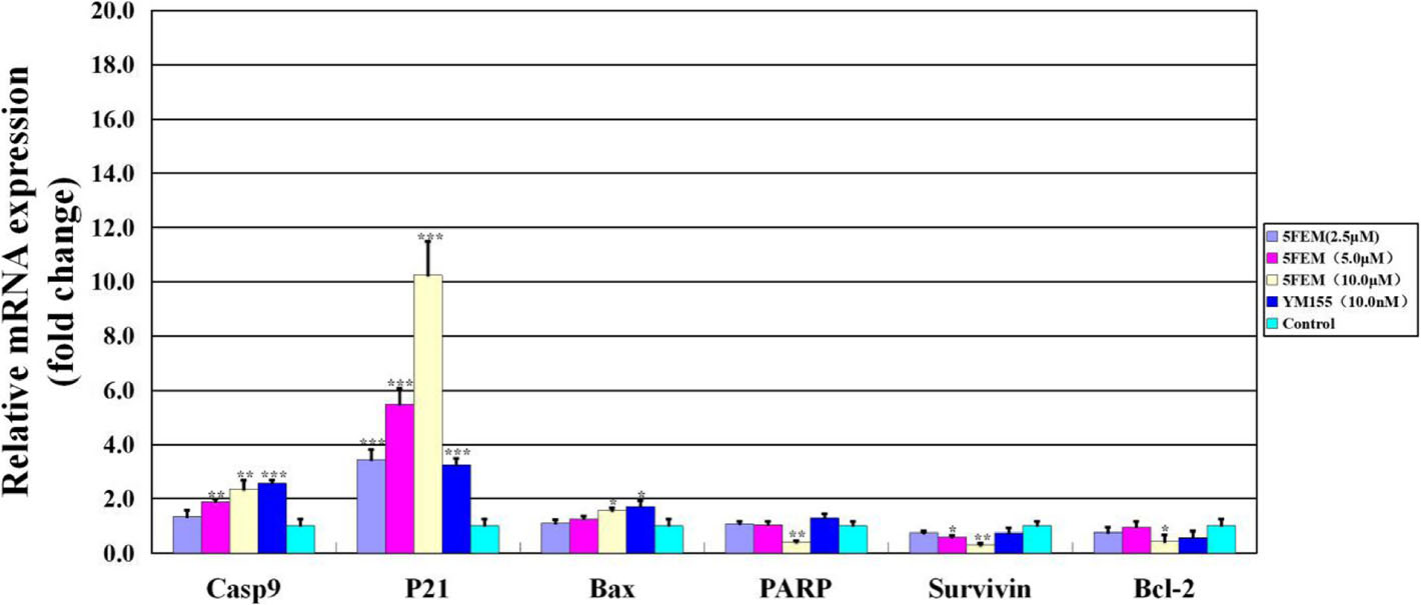
Effects of 5FEM on survivin pathway gene expression of A549-Homo BIRC5 cells

We determined whether 5FEM inhibits A549-Homo BIRC5 cell growth via survivin pathway. The relative expression levels of the survivin-related genes were measured by quantitative real-time reverse transcriptase-PCR (qRT-PCR). As shown in Fig. 10, 5FEM significantly upregulated the mRNA expression levels of caspase-9, P21, and Bax and significantly downregulated those of PARP, survivin, and Bcl-2 in a dose-dependent manner. All gene expression changes contributed to cell apoptosis associated with survivin pathway.

### Expression level of survivin-related proteins

**Fig. 11:**
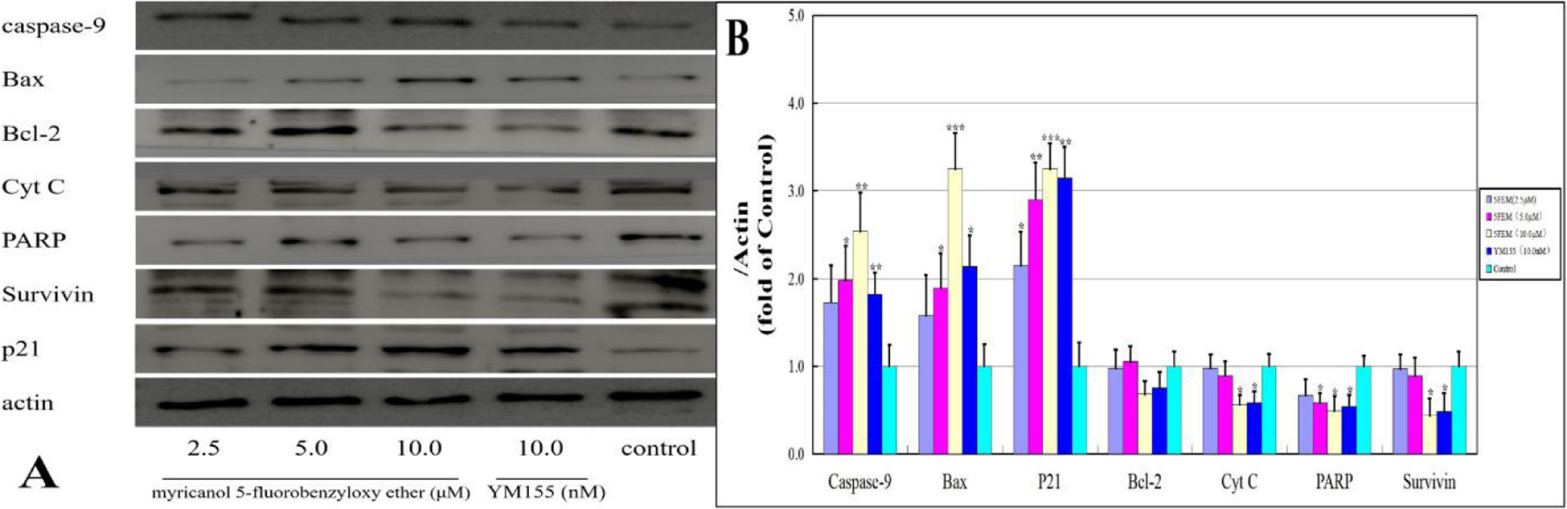
Effects of 5FEM on survive-related proteins in A549-Homo BIRC5 cells. **a** Western blot showed that survive-related proteins expressed in A549-Homo BIRC5 cells. **b** Column figure of protein expression levels in different groups. All data were expressed as mean ± SE of three experiments, and each experiment included triplicate repeats. *p < 0.05, **p < 0.01, and ***p < 0.001 vs. vehicle control

The expression levels of proteins (i.e., caspase-9, Bax, p21, Bcl-2, Cyt C, PARP, and survivin) that are associated with the survivin pathway of apoptosis were measured to elucidate the mechanism involved in the 5FEM-mediated apoptosis of A549-Homo BIRC5 cells further. As shown in Fig. 11a and b, 5FEM could significantly increase the expression levels of the proapoptotic proteins (i.e., caspase-9, Bax, and p21) compared with those of the control group (*p* < 0.01 to *p* < 0.001). The expression level of the antiapoptotic proteins (i.e., Cyt C, PARP, and survivin) was reduced by 5FEM treatment (*p* < 0.05). The changes in these proteins were consistent with the mRNA expression level. These results suggested that 5FEM can induce A549-Homo BIRC5 cell apoptosis through survivin pathway.

## Discussion

The growth characteristics of cancer cells include self-sufficiency in growth signals, insensitivity to antigrowth signals, resistance to cell death, limitless replicative potential, sustained angiogenesis, tissue invasion and metastasis, avoiding immune destruction, tumor promotion inflammation, deregulating cellular energetics, and genome instability and mutation [22]. The role of apoptosis in anti-cancer is becoming increasingly important [23]. Therefore, the induction of tumor cell apoptosis associate with survivin pathway is an important mechanism for targeted cancer therapy [24, 25].

In apoptosis, drugs inhibit the expression levels of survivin and XIAP genes, and Bcl-2 interacting mediator of cell death (BIM) domains of survivin and XIAP proteins can bind to the second mitochondrial activator of caspase (SMAC/DIABLO), antagonize SMAC/DIABLO apoptotic ability, and inhibit activated caspase-3, − 7, and − 9 to promote cell apoptosis [26]. Under the action of drug, the mitochondrial inner membrane pore also opens, thereby triggering mitochondrial membrane permeability transport (MPT) [27]. Apoptosis-inducing factor (AIF) and endonuclease G can be released from MPT, and the translocation of AIF into the nucleus can directly lead to chromatin agglutination. It can directly induce DNA fragmentation, thereby ultimately leading to cancer cell apoptosis.

In Asia, Chinese medicinal herbs have been widely used and are historically well-documented for over hundreds of years. Natural products and their derivatives are attracting attention as drugs for treating various types of cancers, because unlike existing anti-cancer drugs, these substances can exert various anti-cancer effects and have almost no side effects. More than half of known anticancer drugs originated from herbal plants. Natural products remain one of the best reservoirs for new drug discovery [28]. Our research group previous extracted myricanol from the bark of *M. rubra*. Here, we first reported that myricanol can significantly inhibit the growth of A549 cell lines; decrease colony formation; induce A549 cell apoptosis; upregulate caspase-3, caspase-9, Bax, and p21 gene; and downregulate Bcl-2 that is associated with apoptosis gene expression on mRNA and protein level [18, 19]. In the anti-tumor screening of myricanol derivatives, we also used human lung fibroblast 1 (HLF1) as an evaluation of normal cytotoxicity. The results showed that 5FEM was found to have more antitumour activity and low toxicity to HLF1 than that of myricanol and other derivatives [20].

**Fig. 12:**
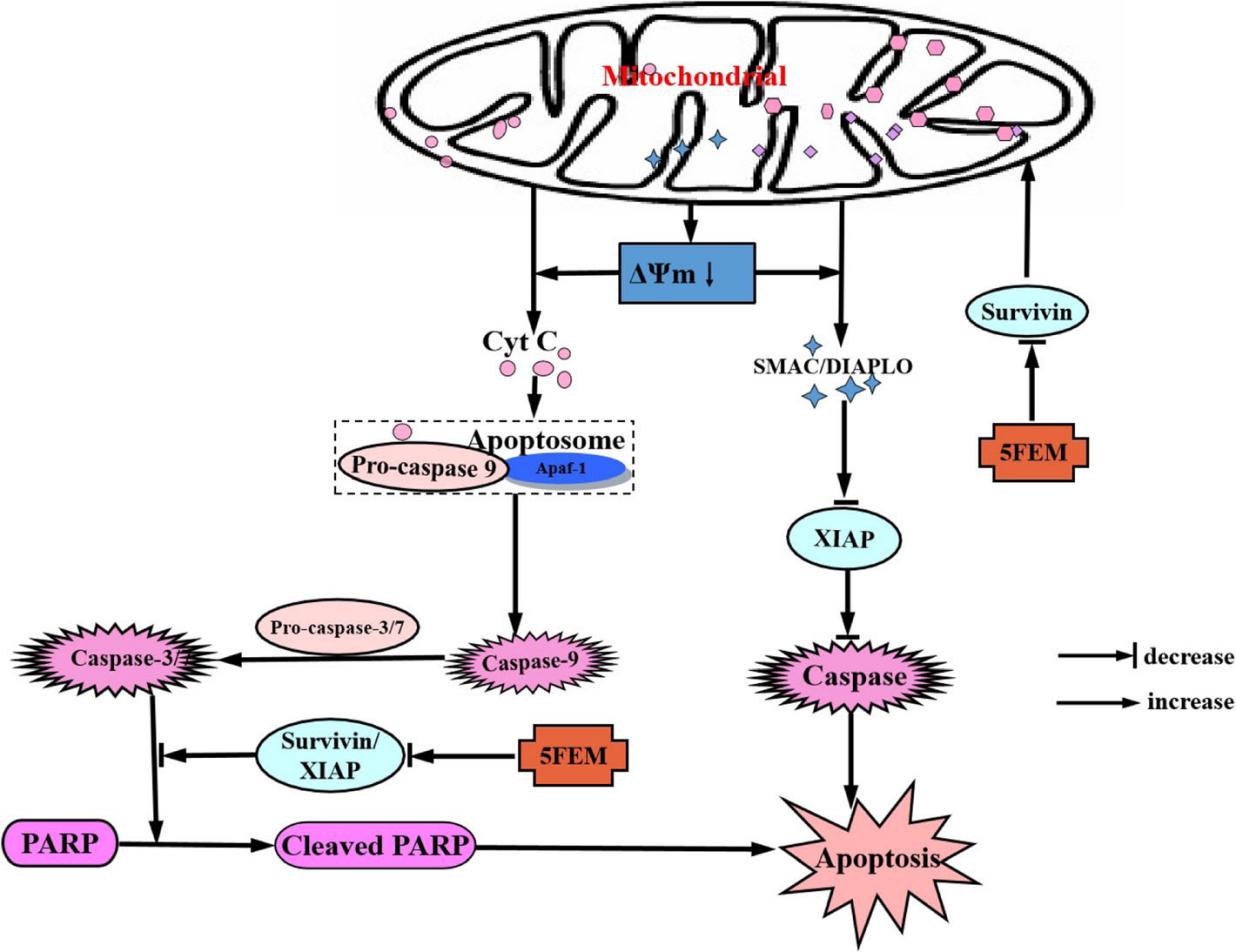
Mechanism of anticancer effects of 5FEM in A549 cells

Combined with the previous research, 5FEM can induces A549 cells apoptosis and inhibits the expression of survivin. We speculated that inhibiting the survivin pathway might be the main antitumor mechanisms of 5FEM (Fig. 12). We selected A549 as the research object. First, A549-Homo BIRC5 cell line with survivin overexpression was constructed. Then, the cytotoxicity, cell apoptosis, cell cycle, ΔΨm, scratch test for cell migration, colony formation, and the expression level of key survivin pathway-related genes were evaluated. However, the further studies (e.g., xenograft animal models and gene knockout experiments using siRNA) should be performed in vivo.

## Conclusions

In summary, the present study showed that 5FEM can significantly inhibit the growth of A549-Homo BIRC5 cells; induce cell apoptosis; increase the G0/G1 population; reduce ΔΨm; inhibit cell migration; inhibit colony formation; upregulate the caspase-9, P21, and Bax expression levels; and downregulate PARP, survivin, and Bcl-2 expression levels. Combined with previous research findings, the current study indicated that 5FEM inhibits human lung adenocarcinoma A549 cells growth by regulation of survivin pathway. These data provided evidence regarding the therapeutic potential of 5FEM as an anticancer drug through the survivin pathway in NSCLC.

## Supplementary information

**Additional file 1.** Original blot images.

## Data Availability

The datasets used and/or analyzed during the current study available from the corresponding author on reasonable request.

## Abbreviations

5FEM: Myricanol 5-fluorobenzyloxy ether
^3^H-TdR: Tritiated thymidine
ΔΨm: Mitochondrial membrane potential
NSCLC: Non-small cell lung cancer
IAP: Inhibitor of apoptosis protein
A549: Human lung adenocarcinoma
DMSO: Dimethyl sulfoxide
7AAD: 7-Amino-Actinomycin D
FBS: Fetal bovine serum
PBS: Phosphate-buffered saline
RPMI-1640: Roswell Park Memorial Institute-1640
FCM: Flow cytometry
YM155: Sepantronium Bromide
PCR: Polymerase chain reaction
LV5-Homo BIRC5: Lentivirus human survivin-overexpression plasmid vector
IC_50_: 50% inhibitory concentration
BIM: Bcl-2 interacting mediator of cell death
SMAC: Second mitochondrial activator of caspase
MPT: Membrane permeability transport
AIF: Apoptosis-inducing factor
