# Supplementary figures and images for: Myricanol 5-fluorobenzyloxy ether regulation of survivin pathway inhibits human lung adenocarcinoma A549 cells growth in vitro
Source: BMC Complement Med Ther. 2020 Sep 3;20:269. doi: 10.1186/s12906-020-03062-8 (PMC7470448; doi:10.1186/s12906-020-03062-8)

Original blot images

| 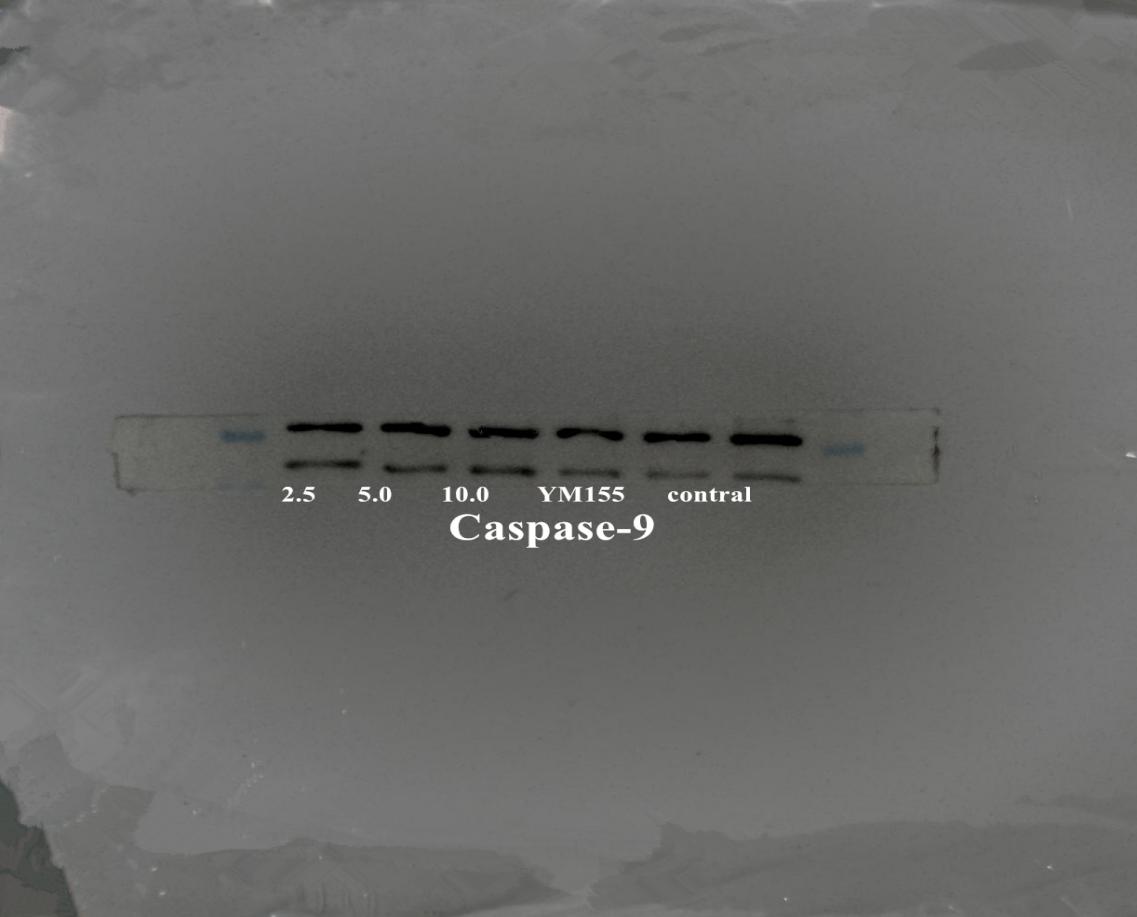 |
| --- |
| 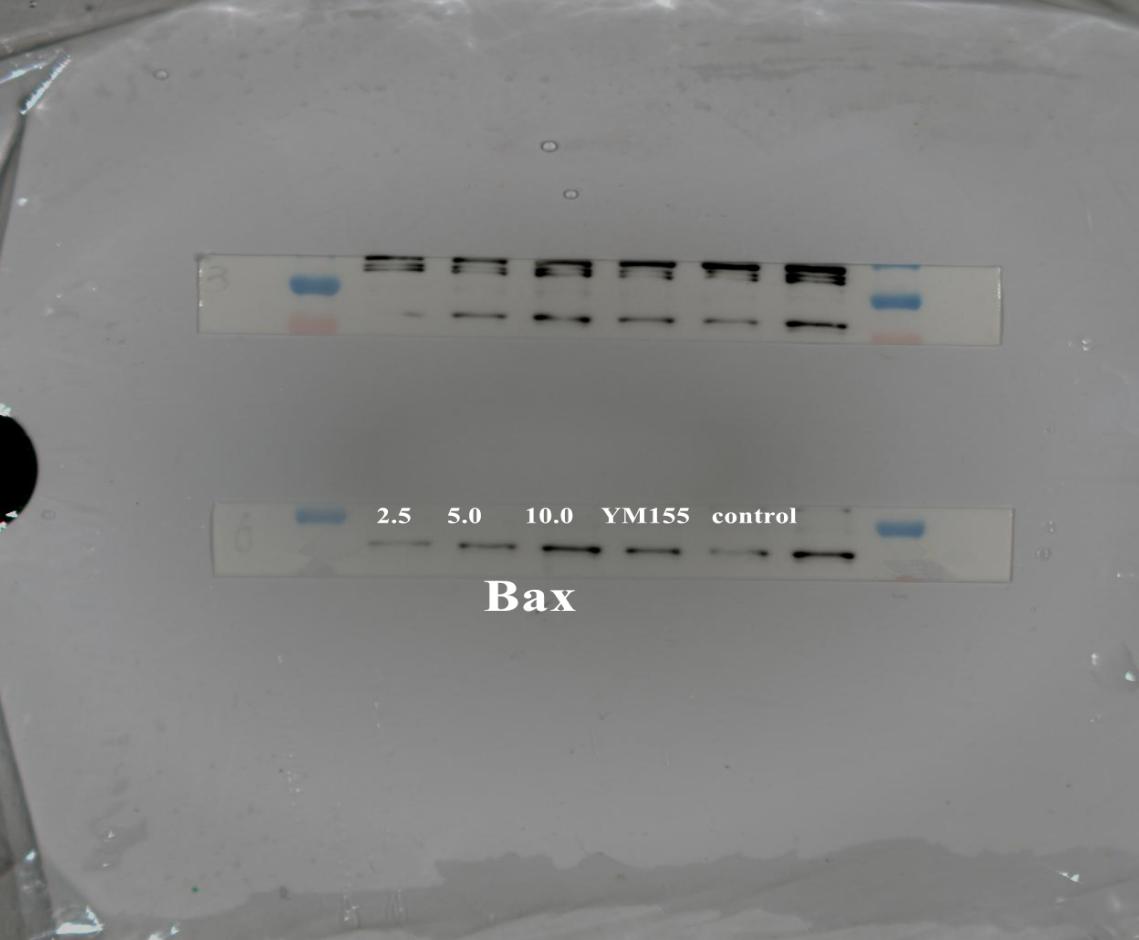 |
| 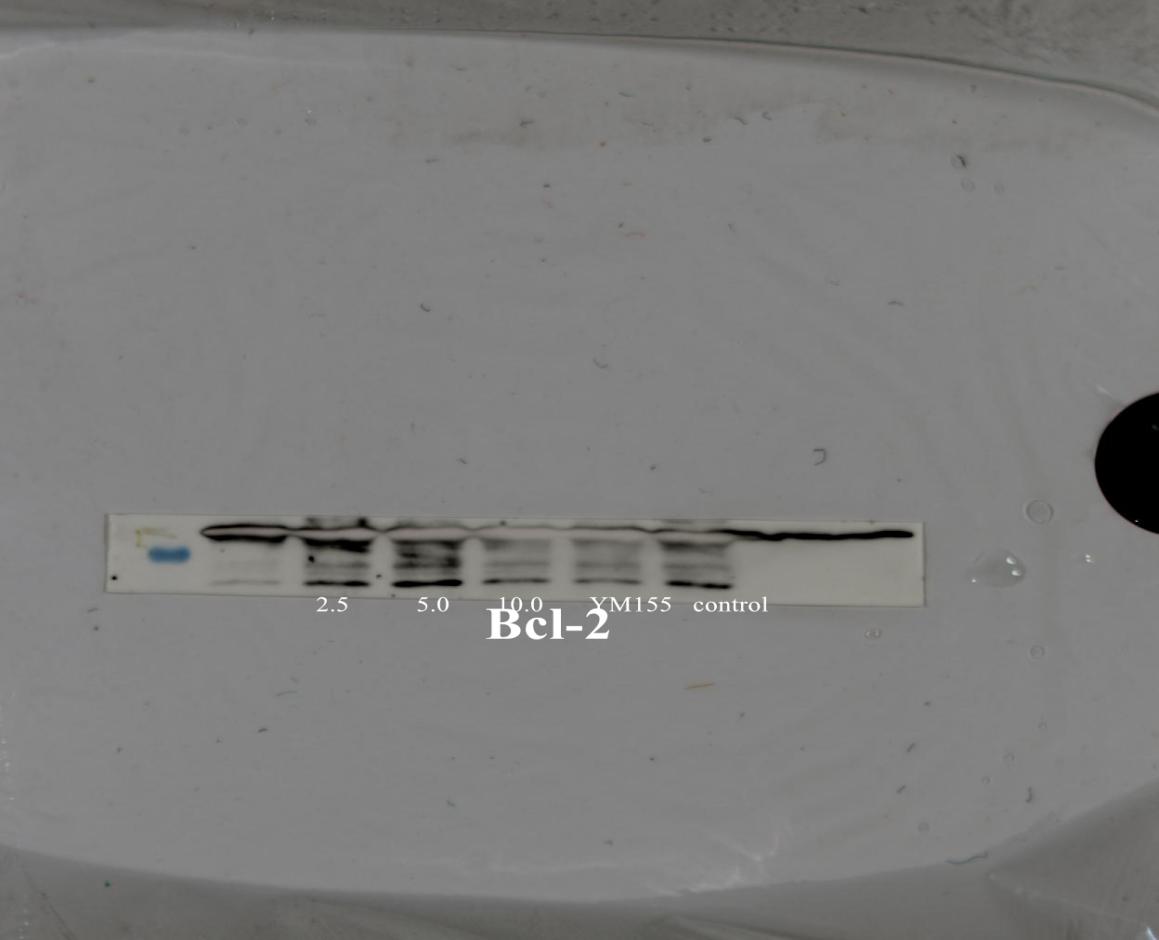 |
| 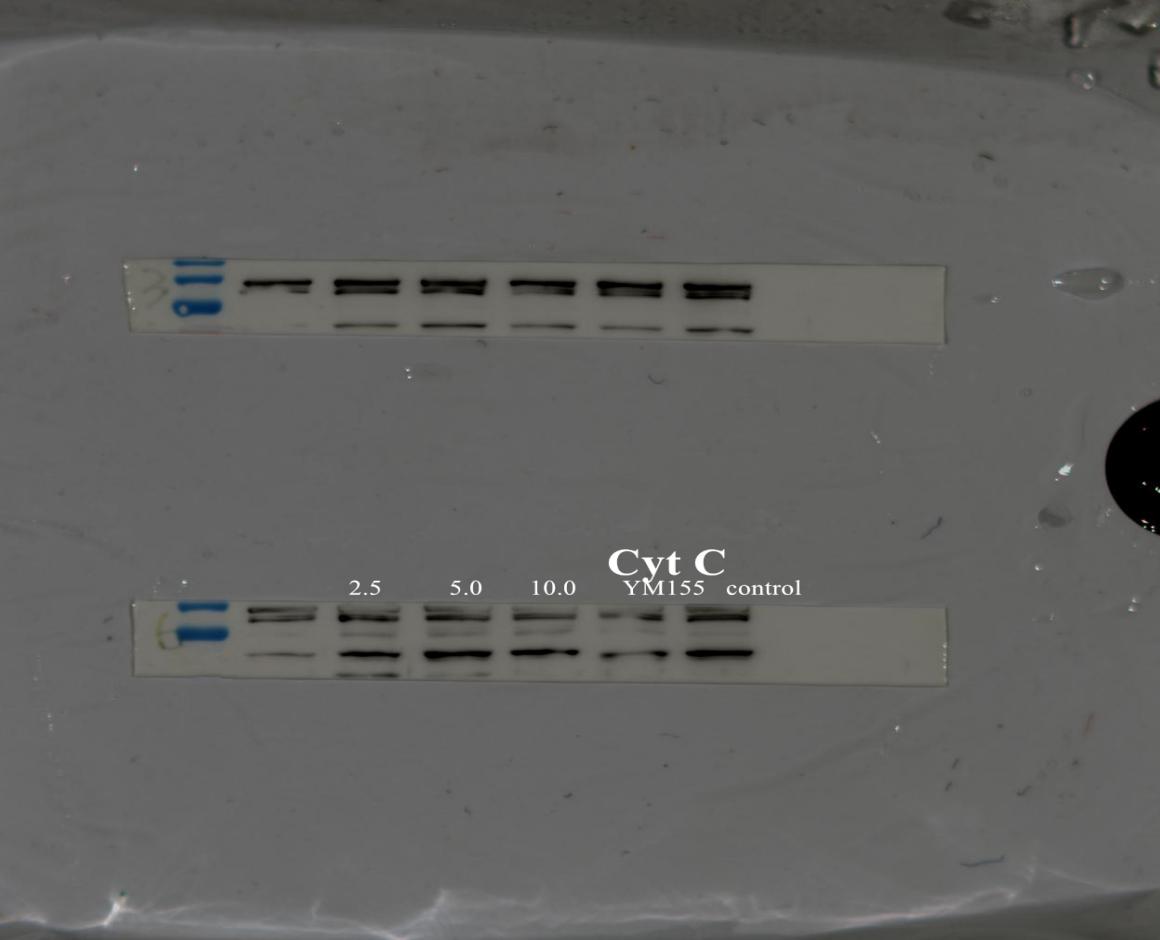 |
| 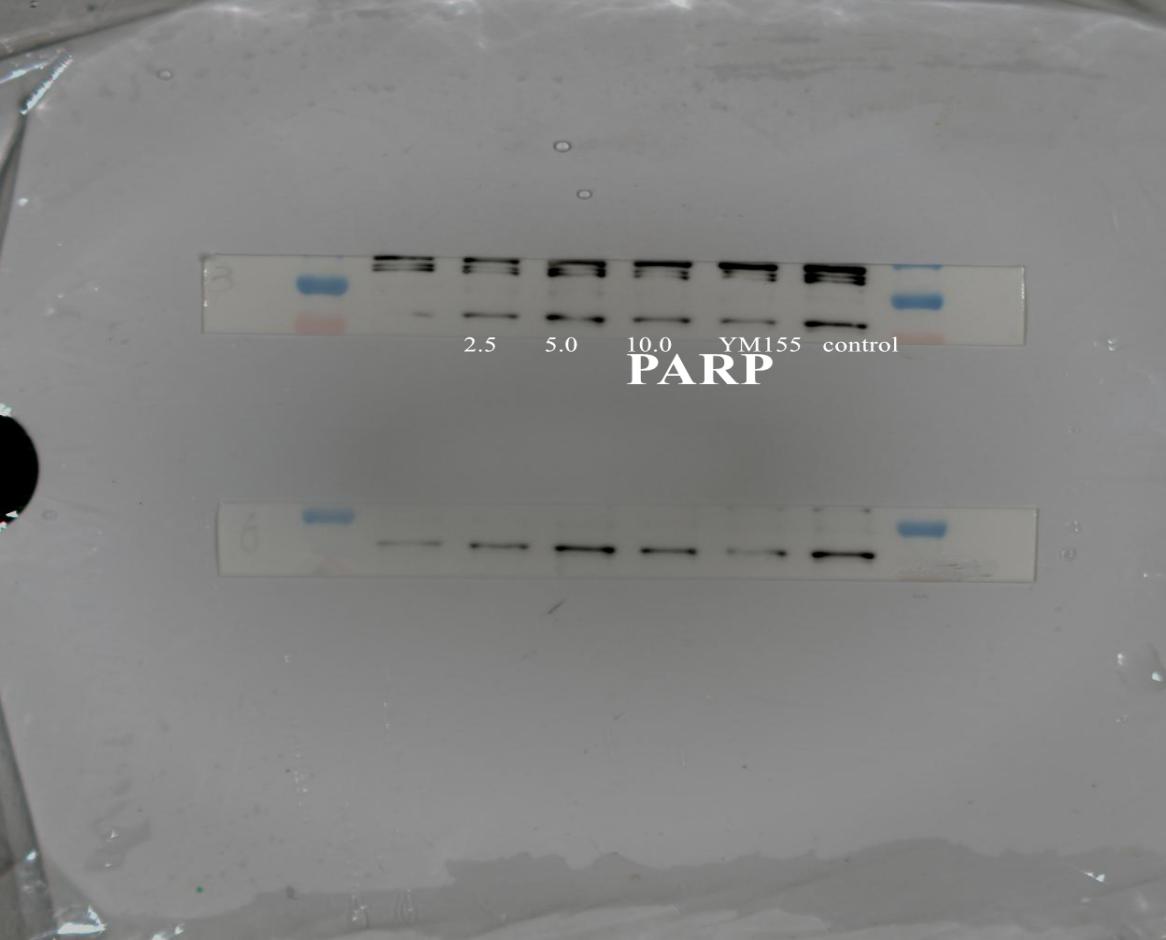 |
| 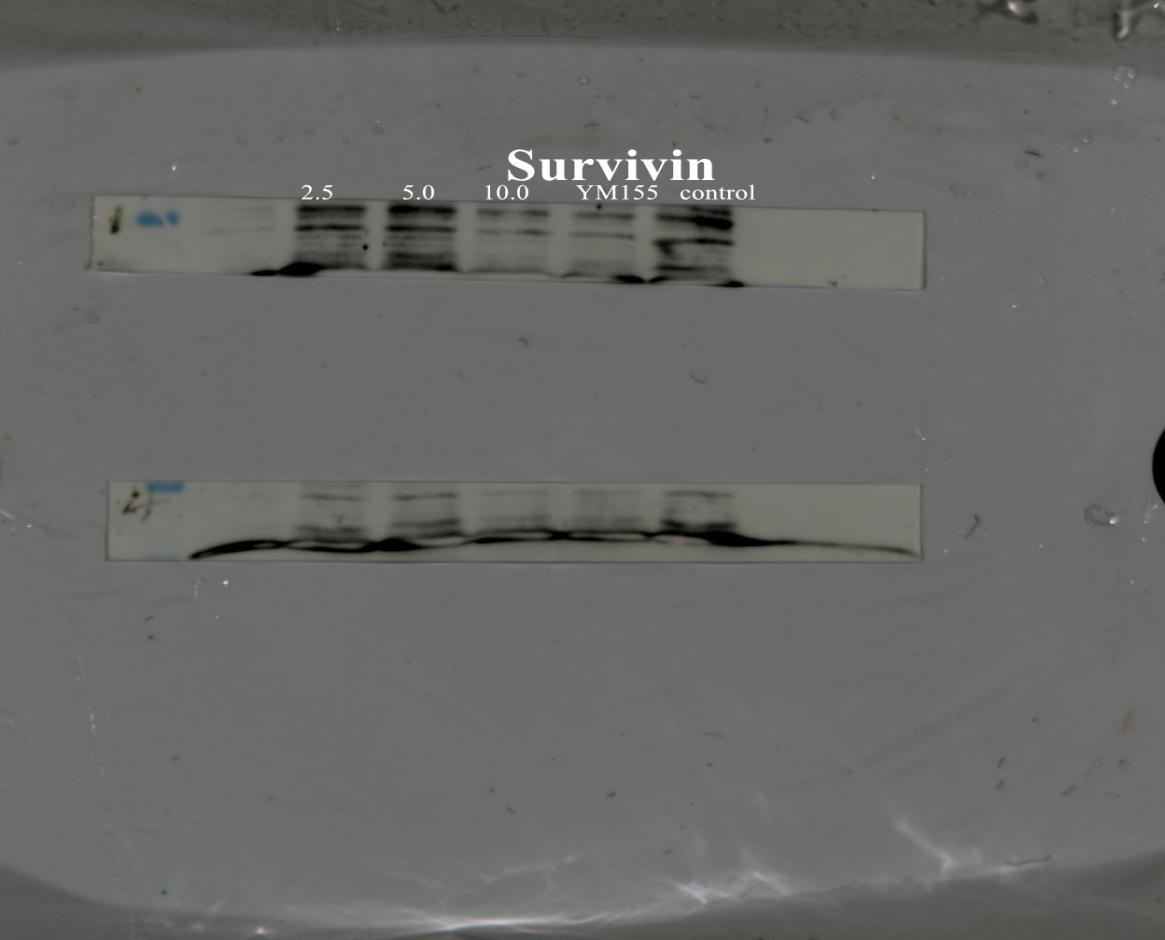 |
| 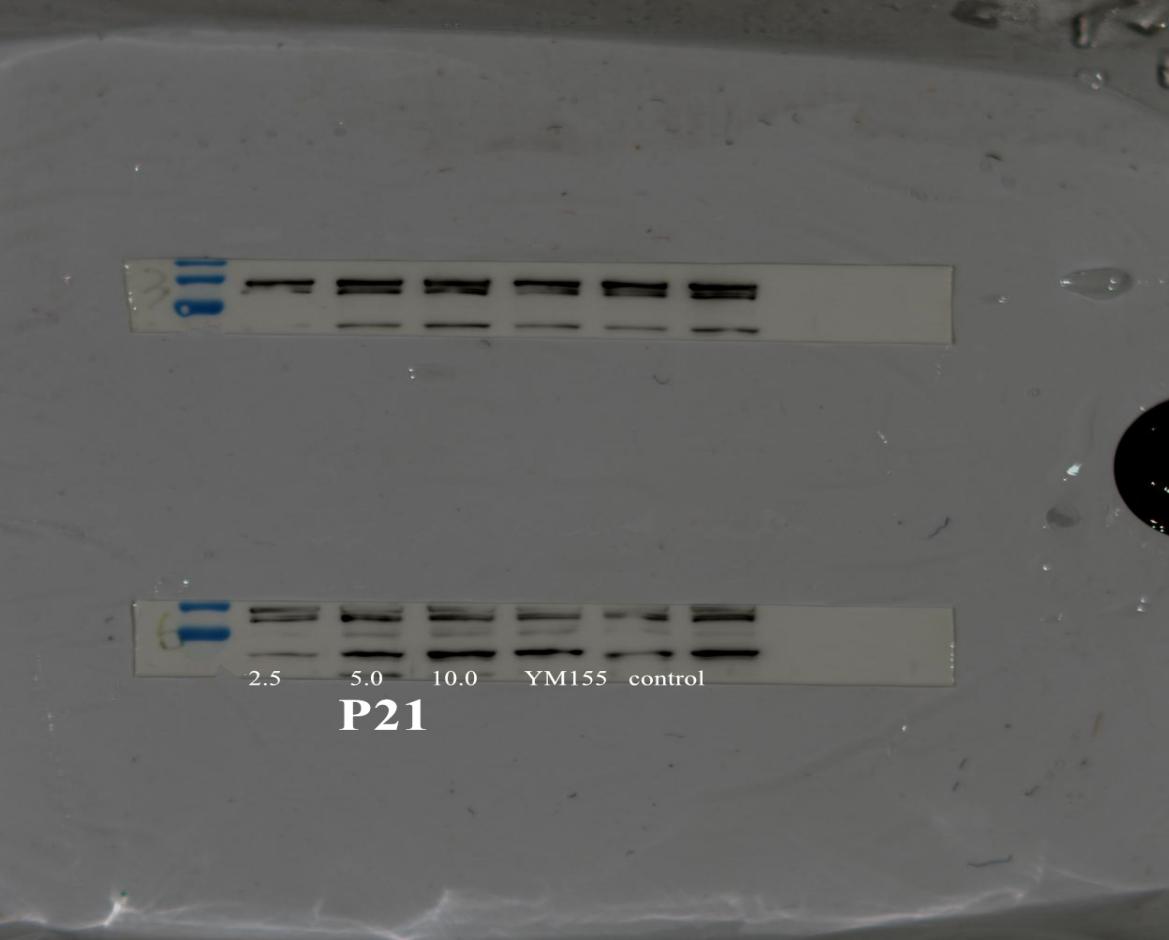 |
| 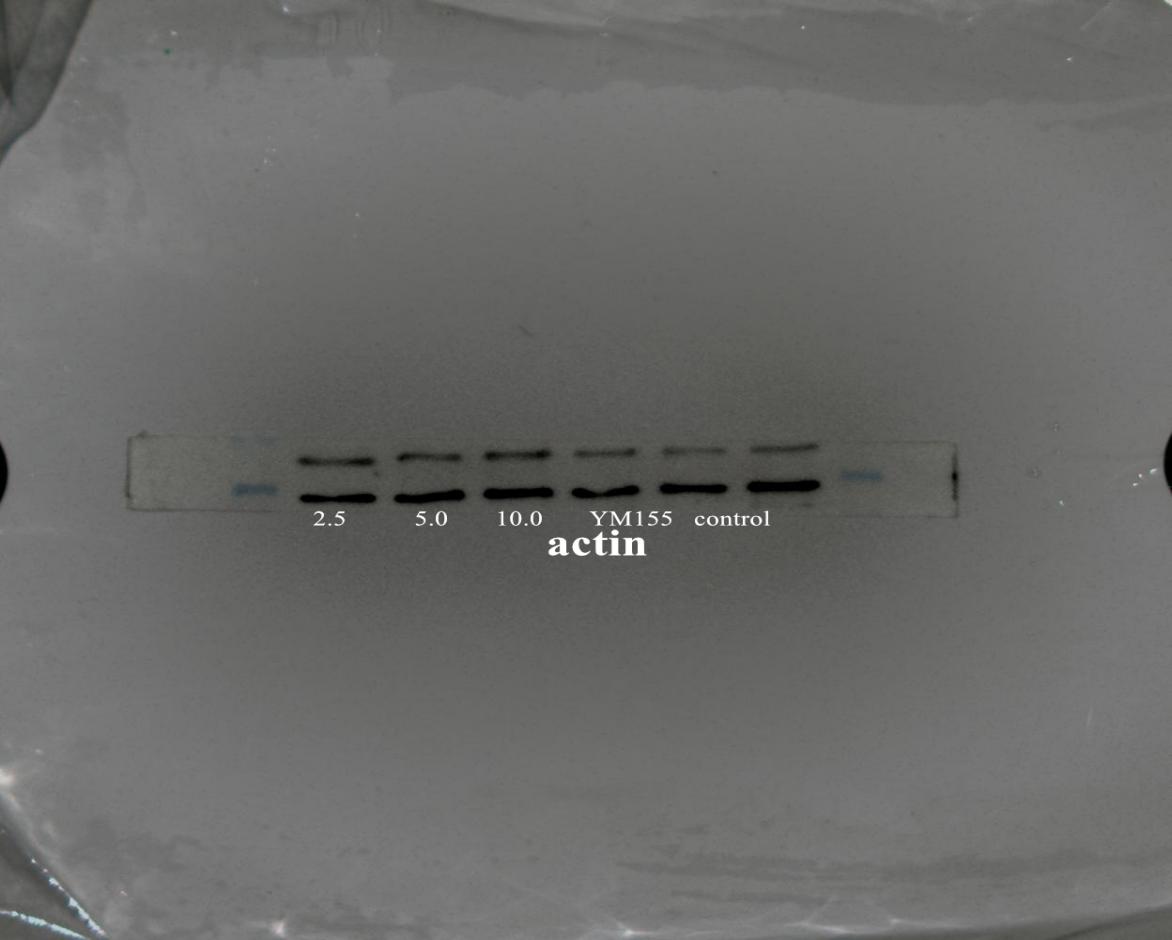 |

Supplement: Supplementary file 1 — Additional file 1. Original blot images. [file 12906_2020_3062_MOESM1_ESM.docx]
